# AGTR1: a potential biomarker associated with the occurrence and prognosis of lung adenocarcinoma

**DOI:** 10.3389/fonc.2024.1441235

**Published:** 2024-10-10

**Authors:** Rui Xiao, Jiajia Han, Yongjian Deng, Ling Zhang, Ying Qian, Nan Tian, Zhen Yang, Lin Zhang

**Affiliations:** College of Life Science/Institute of Molecular Medicine, Zhejiang Chinese Medical University, Hangzhou, Zhejiang, China

**Keywords:** lung adenocarcinoma, bioinformatics, biomarkers, machine learning methods, AGTR1

## Abstract

**Introduction:**

Lung adenocarcinoma, a disease with complex pathogenesis, high mortality and poor prognosis, is one of the subtypes of lung cancer. Hence, it is very crucial to find novel biomarkers as diagnostic and therapeutic targets for LUAD.

**Methods:**

GSE10072 was used for DEGs and WGCNA, and the intersection genes were subjected to enrichment analysis through Metascape and GSEA. Key genes were screened by three machine learning methods. Further, the reliability of key genes was identified by ROC, COX regression analysis and qRT-PCR. CIBERSORT and Spearman analysis were used for understanding the relationships of LUAD, immunity and key genes. In addition, ceRNA networks and potential drugs of key genes were constructed and predicted.

**Results:**

After overlapping 631 DEGs and key module genes, 623 intersection genes were obtained. Subsequently, DUOX1, CD36, AGTR1, FHL5 and SSR4 were further selected using three machine learning methods. Reliability analysis demonstrated that AGTR1 possesses important predictive value for the occurrence and prognosis of LUAD. The enrichment analysis showed that AGTR1 was significantly enriched in the GPCR-related pathways. Immune infiltration analysis showed that the development of LUAD was related to the changes of immune cells such as M2 macrophages and neutrophils, which were regulated by AGTR1. Further, AGTR1 is also involved in regulating immune chemokines, checkpoints and immune regulatory factors such as PECAM1, ADARB1, SPP1 and ENO1, all of them playing important roles in immune cell regulation, tumor cell proliferation and migration. Further, the drug-gene interaction network screened out 13 potential drugs such as Benazepril, Valsartan, Eprosartan, and so on.

**Discussion:**

AGTR1 is a potential biomarker for the occurrence and progression of LUAD, closely related to tumor immunity, proliferation and migration. It can serve as a new target for the diagnosis and treatment of LUAD.

## 1 Introduction

Lung cancer is one of the most highly incident and lethal malignancies globally, with statistics showing that about 1.8 million people die from lung cancer each year (1). Lung cancer is divided into two major types: small cell lung cancer (SCLC) and non-small cell lung cancer (NSCLC), with NSCLC accounting for approximately 85% of all lung cancer patients (2). Early treatment strategies for NSCLC preferentially involve anatomical lung resection combined with lymph node dissection. However, the postoperative cure rate and survival rate remain low, with 30% to 70% of postoperative patients experiencing tumor recurrence or metastasis and the therapeutic effect being very unsatisfactory (3). Lung adenocarcinoma (LUAD) is the most common subtype of NSCLC, accounting for over 40% of all lung cancer cases, and is highly heterogeneous (4, 5). LUAD has a long latency period, with mild early symptoms that are difficult to detect, but with a high incidence of metastasis and malignancy. It is usually diagnosed in the later stages, with a lost opportunity for treatment. Despite there is considerable progress in the treatment of LUAD for the past few years, from traditional radiotherapy and chemotherapy to emerging immunotherapy, the lack of systemic treatment and resistance to radiotherapy and chemotherapy result in a low survival rate of around 15% for all patients with LUAD (4, 6, 7). Due to our incomplete understanding of the pathogenesis and dynamic fluctuations in the tumor gene expression profile, progress in LUAD treatment has reached a plateau and is in urgent need of a breakthrough (8). Therefore, providing new cancer treatment measures and potential biomarkers for LUAD is of great significance.

The gene expression levels can reveal the status of various diseases, including LUAD, and serve as important indicators for basic diagnosis (9). In this study, we employed bioinformatics techniques to integrate genes associated with LUAD and patient sample data from Gene Expression Omnibus (GEO) and The Cancer Genome Atlas (TCGA) for analysis. We used weighted Gene Co-expression Network Analysis (WGCNA) and machine learning methods to identify biologically significant biomarkers. Subsequent validation and functional pathway enrichment were conducted to verify the key genes. Next, qRT-PCR was used to verify differences in the expression of biomarkers in normal lung epithelial cells and LUAD cells. The aim is to seek new diagnostic biomarkers for LUAD diagnosis and treatment. Additionally, immune infiltration analysis was employed to explore the relevance between diagnostic biomarkers and immune cells, immune checkpoints, and immune chemokines, aiming to acquire a more profound comprehension of the immune mechanisms implicated in the progression of LUAD. Furthermore, a competitive endogenous RNAs (ceRNAs) network was established to elucidate the regulatory mechanisms of diagnostic biomarkers in LUAD. Lastly, we conducted potential drug predictions to identify effective drugs that may have a positive impact on LUAD.

## 2 Materials and methods

### 2.1 Data acquisition and analysis

**Figure 1:**
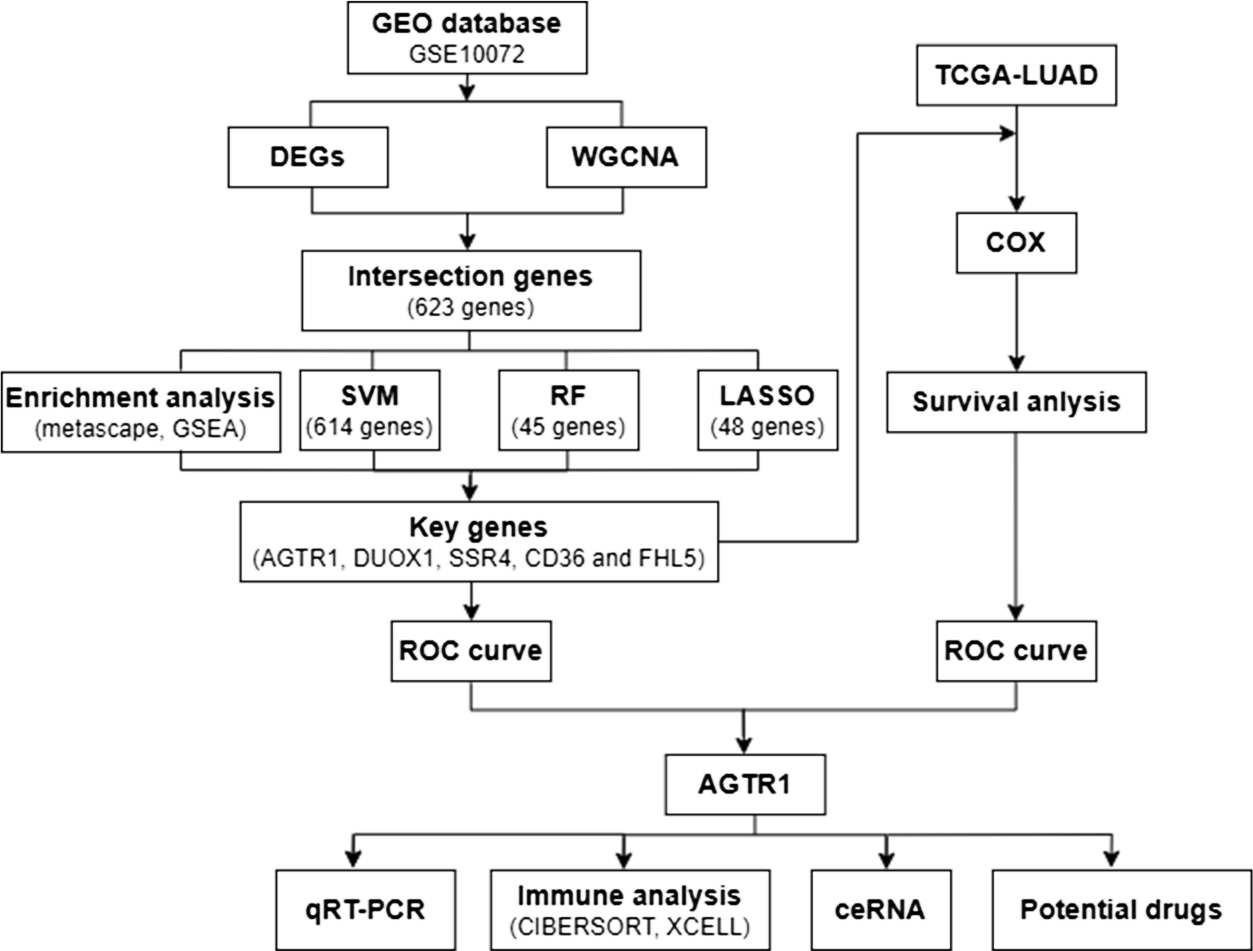
Program flowchart for this study.

**Figure 1** shows the analysis process of this study. The GEO (https://www.ncbi.nlm.nih.gov/geo/) database includes gene expression and regulation data from various species, including microarray chips, RNA sequencing (RNA-seq), and other high-throughput sequencing technologies. We downloaded the Series Matrix File GSE10072, which is a dataset with the richest number of LUAD samples and the highest number of detected genes, from GEO database using “lung adenocarcinoma” as the keyword. GSE10072 includes a total of 107 samples (58 tumor samples and 49 normal samples) and 13227 gene expression data. The specific clinical information of the sample showed in **Supplementary Table S1**. After performing analysis and processing on the data, we obtained differentially expressed genes (DEGs). The selection is performed based on a P value less than 0.05 and an absolute value of log fold change (|log2FC|) greater than 1. Moreover, volcano plot and heatmap were created using the above data to visualize the differential expression of DEGs. Additionally, we accessed TCGA database (https://portal.gdc.cancer.gov/), which is a comprehensive genomics database focused on various types of cancer. It is a project based on high-throughput sequencing, genomic analysis, and clinical data, aimed at understanding the molecular characteristics, pathogenic mechanisms, and therapeutic potential of cancer. We obtained a total of 403 LUAD samples, 48 normal samples and 15647 gene expression data from the TCGA database. The patient clinical information related to LUAD can be found in **Supplementary Table S2**. The data obtained from TCGA will be subsequently used for COX proportional hazards regression analysis (COX).

### 2.2 Construction of co-expressed gene modules

WGCNA is a method used for analyzing gene co-expression networks. It involves calculating the co-expression patterns between genes and clustering genes with related functions or involvement in the same biological processes into functional modules. By studying these modules, we can discover module genes closely related to LUAD and understand their positions and functions within the network. We used R package of WGCNA to build a co-expression network for all genes from GSE 10072. The soft threshold of GSE 10072 dataset was set as 8. The weighted adjacency matrix was converted into a Topological Overlap Matrix (TOM) to appraise network connectivity, and a clustering dendrogram was constructed by a hierarchical clustering method based on the TOM matrix (10). The genes were assorted into different categories based on their weighted correlation coefficients and the similar expression genes were grouped into identical modules. The gene modules highly correlated with LUAD were identified and selected through Pearson correlation analysis. Then, the intersection genes obtained by taking the overlap between DEGs and module genes using a Venn diagram.

### 2.3 Gene function annotation and enrichment analysis

We performed enrichment analysis and functional annotation of genes by accessing the Metascape analysis website (11) (http://metascape:gp/index.html#/main/step1), and the Gene Set Enrichment Analysis (GSEA) official website (12) (http://software.broadinstitute:gsea/index.jsp). Metascape is an online tool for gene functional annotation and enrichment analysis, used to interpret results from genomics, transcriptomics, and proteomics research. The intersection genes were submitted to Metascape analysis website for GO and KEGG analysis. The p-value < 0.01 was considered significant.

GSEA is a commonly used method for gene enrichment analysis and assesses the enrichment level of a gene set in a predefined biological process, signaling pathway, or disease by comparing its relevance to a particular biological process. By conducting GSEA analysis and applying a filtering criterion of FDR<0.25 and P<0.05, we identified significantly enriched pathways.

### 2.4 Selection and validation of key genes

Intersection genes were further sifted for key genes related to LUAD through random forests (RF), least absolute shrinkage and selection operator (LASSO) logistic regression, and support vector machine-recursive feature elimination (SVM-RFE) (9). Among them, LASSO belongs to the linear regression algorithm, which can establish a relationship model between two or more variables, and predict the values of one or more continuous variables by fitting the data. RF and SVM belong to classification algorithms that can maximize correct classification on training data and have good generalization ability on test data by finding the optimal decision boundary. Within them, RF can effectively reduce overfitting by constructing a large number of decision trees and average their results, especially when dealing with high-dimensional data, and SVM-RFE can find the optimal separating hyperplane for data and effectively perform complex classification tasks. The “randomForest” R package was utilized to implement the random forest technique, the “glmnet” package in R was utilized for LASSO logistic regression analysis, and we used the R package “e1071” for SVM-RFE in this study. Then, we obtained key genes by taking the intersection genes acquired via three different machine learning methods.

The key genes were evaluated based on the analysis of receiver operating characteristic (ROC) curves, and the area under the curve (AUC) was calculated to evaluate the predictive capability of a binary classification model (9). ROC analysis is also employed to compare the predictive ability of key genes and clinical factors for prognosis. Using the sample data obtained from GEO, ROC curves of smoking and key genes were plotted by the “pROC” R package. Afterwards, COX regression analysis was performed by “survival” package on the selected data based on TCGA database. Drawing survival curves used “survminer” package, and “survivalROC” package was used to draw survival curves specifically for ROC. The Cox regression model can estimate the hazard ratio and its statistical significance for predictive factors on event occurrence. Finally, we used “ggplot2” and “ggpubr” packages to draw a box plot to display the expression differences of key genes in the sample data obtained from TCGA. Through these analyses, we can compare and identify the key genes with the strongest correlation to LUAD.

### 2.5 Validation of key gene by qRT-PCR

QRT-PCR was performed to confirm the expression of key gene in the normal control (NC) and LUAD groups. Normal lung epithelial cells BEAS-2B cells and LUAD epithelial cell lines (A549, H1299 and H1975) were acquired from the Cell Bank of the Chinese Academy of Sciences (Shanghai, China). Among them, A549 was taken from primary LUAD tissue of a 58 years old white male. H1299 was derived from a 43 years old white male LUAD patient with lymph node metastasis. And H1975 was isolated from cancer tissue of a white female patient with primary LUAD. These cells were cultivated at 37°C and 5% CO2 and in DMEM and RPMI-1640 (Gibco, USA) supplemented with 1% penicillin–streptomycin (Yeasen, Shanghai, China) and 10% fetal bovine serum (Gibco, USA). All dissected samples were immediately stored in liquid nitrogen until they were prepared for total RNA extraction (13).

Total cellular RNA was extracted by TRIzol (Invitrogen, Carlsbad, CA, USA). We reverse transcribed RNA to cDNA using a reverse-transcription kit (Vazyme, Nanjing, China). QRT-PCR was performed with the SYBR Green Master Mix Kit (Vazyme, Nanjing, China) on an iQ5 real-time PCR machine (Bio-Rad, Hercules, CA, USA). All expression levels are based on internal control (β-Actin). The qRT-PCR cycles were as follows: step1, preparative denaturation (10 min at 95 °C); step 2, 40 cycles of denaturation (10 s at 95°C) and annealing (20 s at 60 °C); and step 3, dissociation, following the manufacturer’s protocol (13). Forward primer of hub gene was as followed: 5′-CTGCTATGCCCATCACCATCTG-3′; reverse primer of hub gene was as followed: 5′-GATAACCCTGCATGCGACCTG-3′. The 2–ΔΔct method was used to calculate relative gene expression levels.

### 2.6 Immune cell infiltration analysis

Based on gene expression data, CIBERSORT is a method to evaluate the relative abundance of different cell types within tumor tissues (13–15). It applies support vector regression principles to deconvolute the expression matrix of immune cell subtypes (13, 16). XCell is a powerful algorithm designed to assess the infiltration levels of 64 distinct types of stromal and immune cells, encompassing cellular components such as extracellular matrix cells, epithelial cells, hematopoietic progenitors, as well as both innate and adaptive immune cells. The CIBERSORT was utilized to analyze the data of LUAD samples, allowing for estimation of the associated proportions of 22 distinct immune-infiltrating cells. Spearman analysis was implemented to explore the association between gene expression and immune cell content. We also analyzed the correlation between key genes and immune stroma and immune microenvironment using xCell. Furthermore, we conducted correlation analysis for key genes and immune chemokines, immune checkpoints as well as other immune regulatory molecules.

### 2.7 Construction of ceRNA regulatory network

An increasing amount of researches suggests that long non-coding RNAs (lncRNAs) function as ceRNAs by competing for binding to microRNAs (miRNAs), thus exerting a regulatory effect on target genes expression (2). We predicted target miRNAs of key genes through the databases miRTarBase (https://mirtarbase.cuhk.edu.cn/). Next, the miRNet (https://www.mirnet.ca/miRNet/home.xhtml) database was used to predict the possible lncRNAs that are targeted by the miRNAs. Subsequently, A ceRNA regulatory network was constructed by integrating the interaction between miRNAs and lncRNAs. The ceRNA network was visualized using OmicStudio (https://www.omicstudio.cn/home).

### 2.8 Prediction of potential drugs

To explore drug-gene interactions, we applied the Drug-Gene Interaction database (DGIdb) (https://dgidb.org) and Comparative Toxicogenomics Database (CTD) (http://ctdbase.org/) to forecast prospective drugs for the treatment of LUAD, based on key gene relative with LUAD. The data was shown via Cytoscape software.

### 2.9 Statistical analysis

Statistical analysis was executed using R package (version 4.3.1). Two-tailed tests were used for all data validation, and a significance level of P < 0.05 or P<0.01 were considered statistically significant. The relevance analysis was performed by either Pearson correlation or Spearman correlation, depending on the specific situation.

## 3 Results

### 3.1 DEGs Screening in lung adenocarcinoma

**Figure 2:**
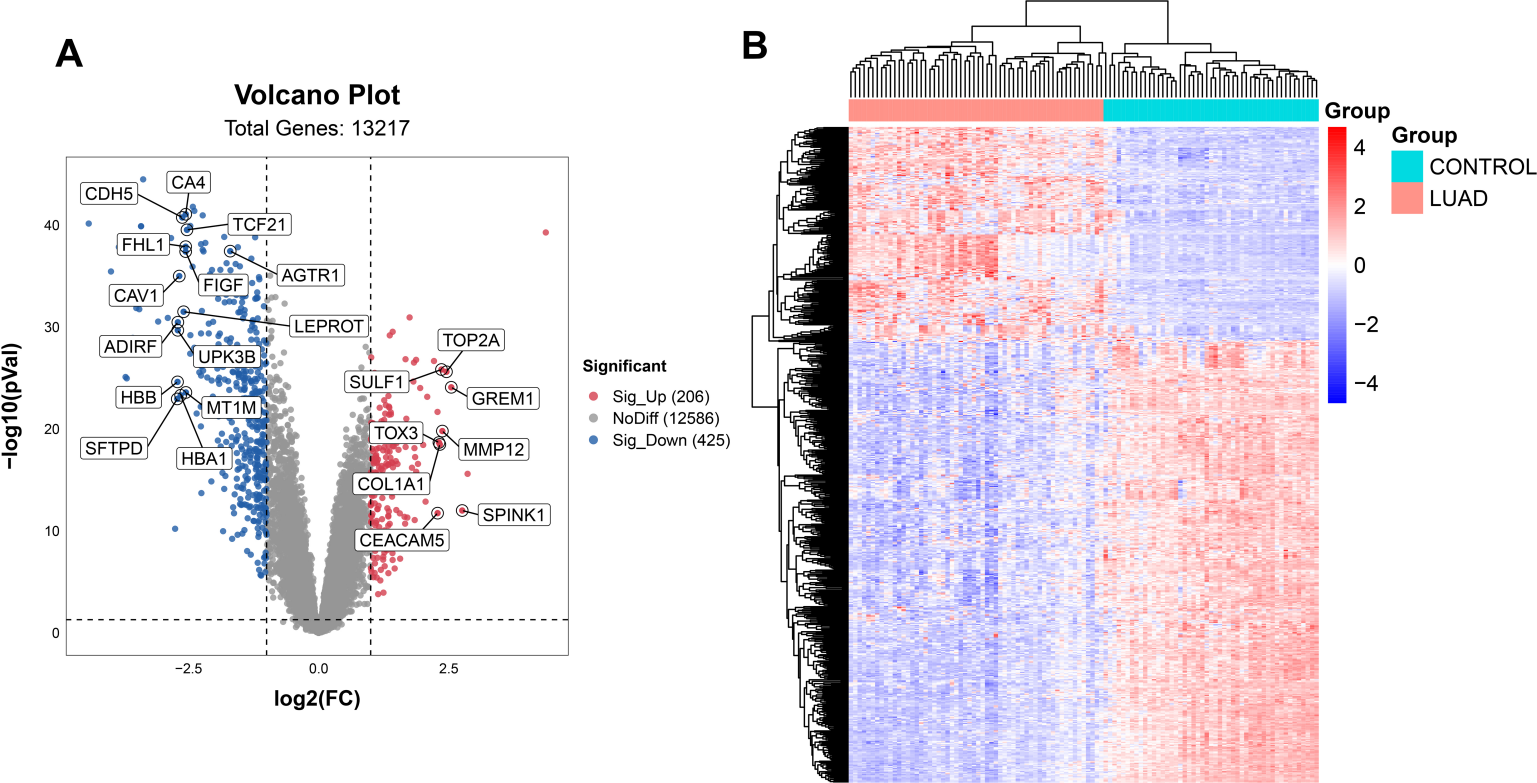
Acquisition of DEGs. The DEGs were shown on the volcano plot **(A)** and the heatmap **(B)** for the GSE10072 datasets.

First, we extracted gene expression data from the dataset GSE10072 in GEO database for subsequent analysis. Then, we performed sample grouping using GEO2R and conducted DEG analysis on the samples to identify 631 DEGs, including 206 upregulated and 425 downregulated genes. A volcano plot was constructed by the “ggplot2” package (**Figure 2A**) and a heatmap was generated using the bioinformatics cloud platform (http://www.bioinformatics.com.cn) (**Figure 2B**) to visualized the DEGs.

### 3.2 Identification of co-expression gene modules in lung adenocarcinoma

**Figure 3:**
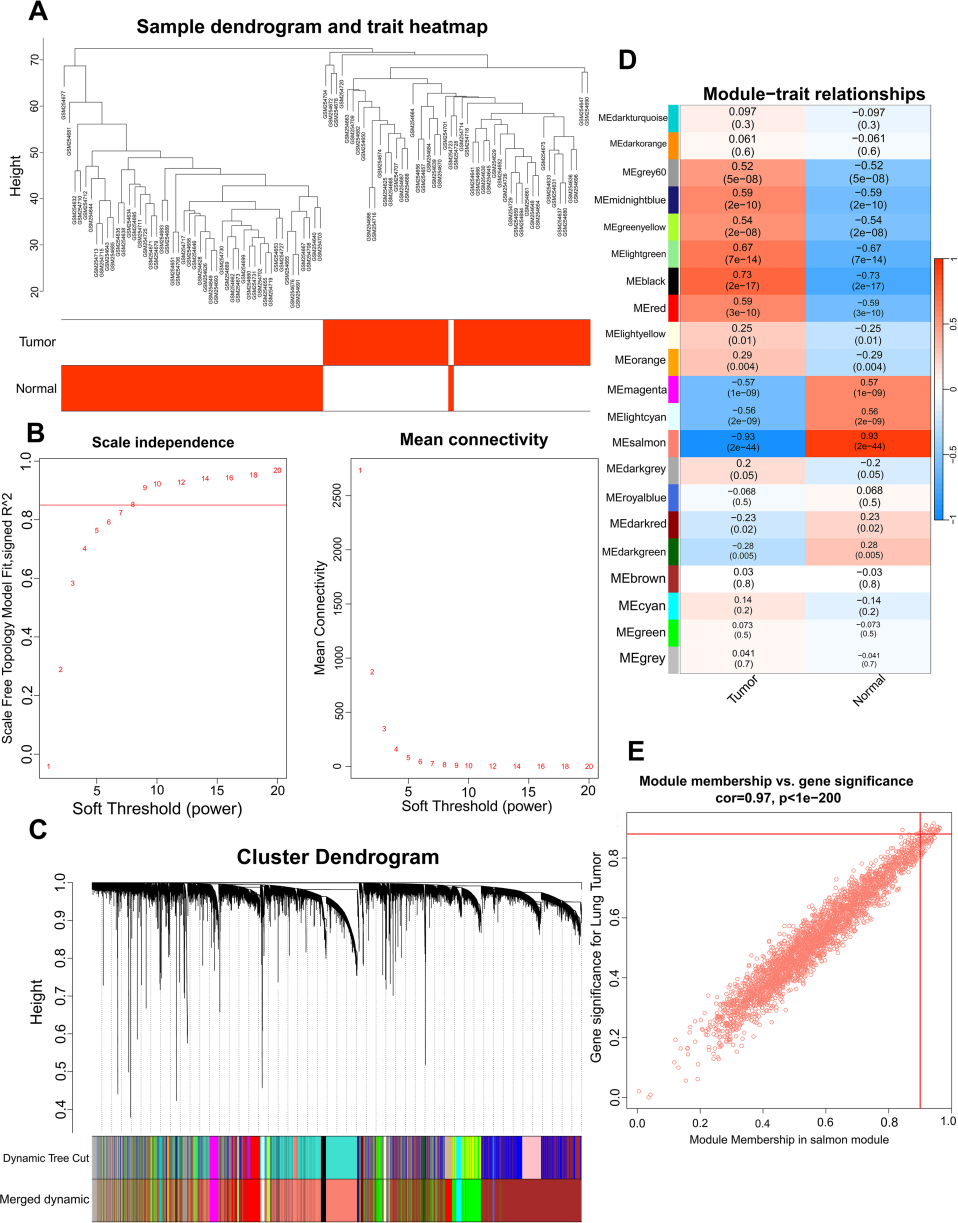
Identification of the hub module via WGCNA. Sample dendrogram classified by tumor and normal samples **(A)**, scale-free fit index and the average connectivity of soft threshold power **(B)** and hierarchical clustering tree of genes based on topological overlap **(C)** were obtained in GSE10072. The correlation between module genes and LUAD in GSE10072 **(D)**. A scatter plot of the salmon module in GSE10072 **(E)**.

WGCNA was employed in GSE10072 dataset to identify gene modules characterized by co-expression of multiple genes. The phenotypic data of samples obtained from GSE10072 dataset were collated, and a cluster tree divided into tumor group and normal group was obtained (**Figure 3A**). We selected a soft threshold of 8 for constructing a scale-free network (**Figure 3B**). Next, we employed dynamic hybrid cutting to build a hierarchical clustering tree, which resulted in the formation of gene modules (13, 16). Within the tree branches, we observed a cluster of genes exhibiting similar expression profiles (**Figure 3C**). In the clustering tree, the different branches represent distinct gene modules, while the different colors represent different modules. Twenty-one modules were constructed for GSE10072 (**Figure 3D**). The modules in salmon, grey60, midnightblue, greenyellow, lightgreen, black, red, magenta, and lightcyan colors showed significant associations with LUAD and were chosen as the hub modules for further analysis. The scatter plots of module genes most highly associated with LUAD is shown in **Figure 3E**.

### 3.3 Functional enrichment analyses of intersection genes

**Figure 4:**
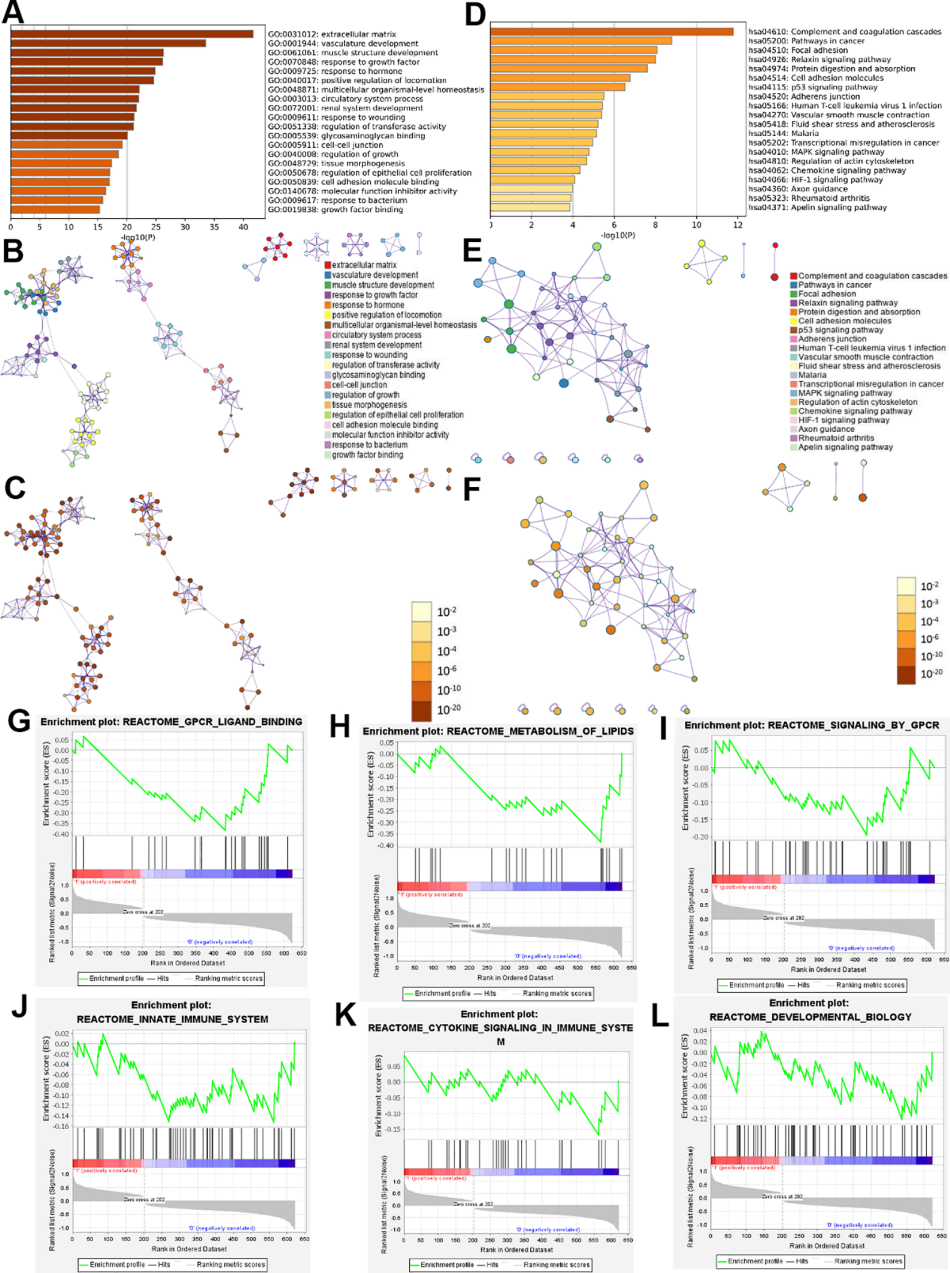
Functional enrichment analysis results of intersection genes. Enrichment analysis by Metascape for intersection genes, including GO and KEGG **(A-F)**. The bar graphs used a discrete color scale to represent statistical significance **(A, D)**. the colors of different points in **(B, E)** represent different biological functions and pathways. In **(C, F)**, the darker colors indicating higher levels of statistical significance. Dot size indicates the degree of enrichment, and the larger the dot, the higher the enrichment level of the gene in **(B, C, E, F)**. GSEA used to analyze and evaluate signal pathways related to intersection genes **(G-L)**.

In order to understand the biological functions of genes, we conducted Metascape analysis on the intersection genes (**Supplementary Table S3**) obtained from DEGs and module genes. GO and KEGG pathway analyses were conducted using Metascape. We sorted the results in ascending order based on the False Discovery Rate (FDR), and select the top 20 biologic functions, respectively. GO analysis revealed that they were implicated in many aspects, including vasculature development, response to growth factor, response to hormone, extracellular matrix, multicellular organismal-level homeostasis, circulatory system process (**Figures 4A–C**). KEGG analysis of intersection genes showed pathways involved Pathways in cancer, Focal adhesion, Cell adhesion molecules, p53 signaling pathway (**Figures 4D–F**).

GSEA was used to analyze and evaluate signal pathways related to intersection genes. We sorted the results in descending order based on the Normalized Overlap Measure p-value (NOMp-val). The signaling pathways included GPCR ligand binding, metabolism of lipids, signaling by GPCR, innate immune system, cytokine signaling in immune system, developmental biology and so on (**Figures 4G–L**).

### 3.4 Identification and validation of key genes

**Figure 5:**
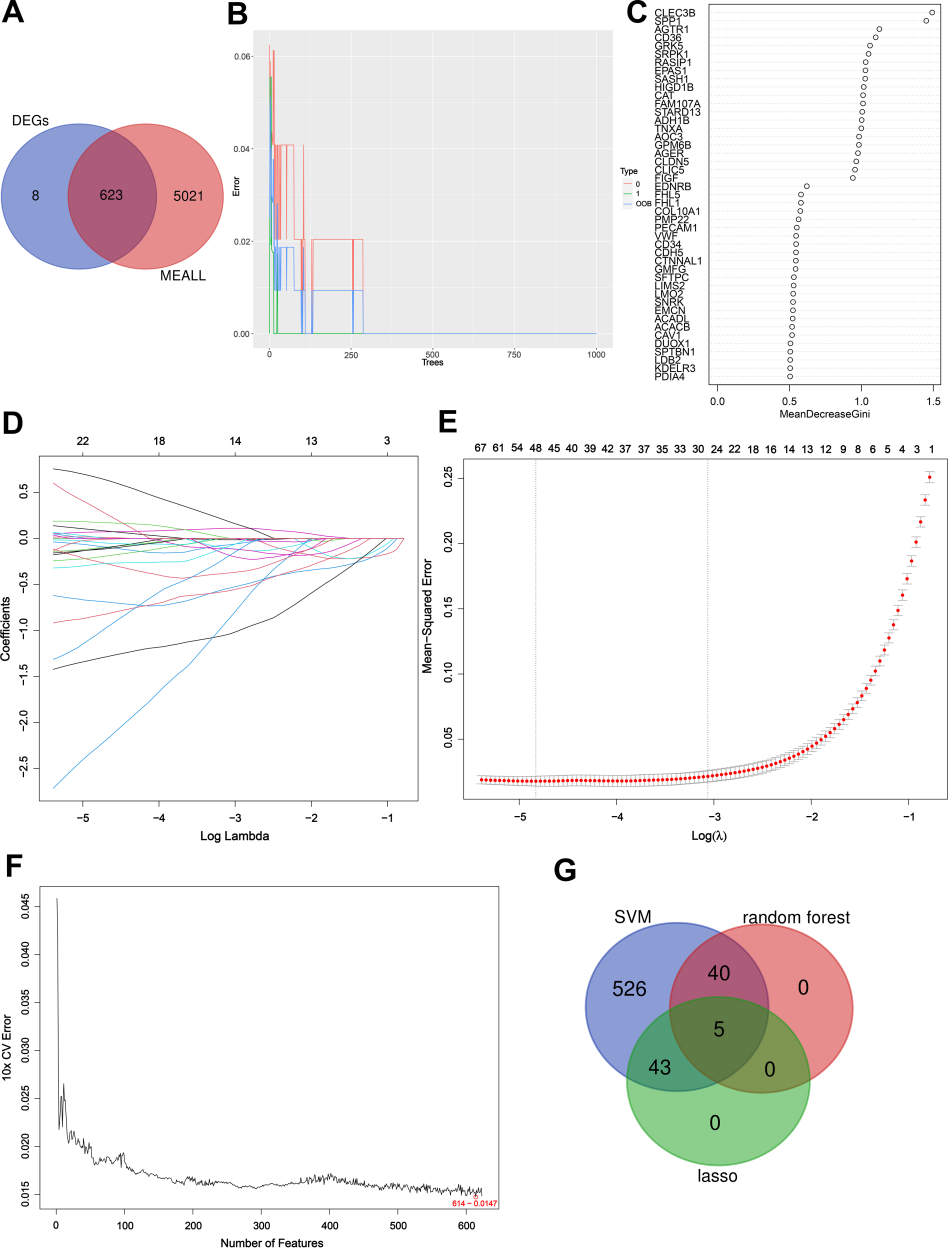
Detection of key genes using three machine learning methods. Venn diagram of key module genes versus DEGs **(A)**. The error rate confidence intervals for random forest model **(B)**. Sorting 45 gene importance using the Gini index **(C)**; As the lambda value varies, the variables whose coefficients are compressed to zero later are considered more important **(D)**. The mean squared error plot of a LASSO model with error bars representing standard errors showed 48 genes were obtained **(E)**; Based on SVM-RFE to screen 614 key genes **(F)**. Three machine learning approaches to obtain the intersection of genes and obtain key genes **(G)**.

By Venn diagram (**Figure 5A**), we obtained the same region between DEGs and hub module genes, and identified 623 intersection genes. We utilized RF in conjunction with feature selection to establish the relationship between the error rate, the number of classification trees, and the genes sorted in descending order of relative relevance (9) (**Figure 5B**). Using RF, we respectively identified sets of 45 genes (**Figure 5C**). Based on the optimal lambda value of 0.007991898, a Lasso regression model is constructed. We employed LASSO regression analysis to choose 48 predicted genes out of the univariate variables that demonstrated statistical significance (**Figures 5D, E**). SVM-RFE used an iterative process to construct an SVM model and calculate the relevance ranking of genes associated with LUAD in order to select the most important 614 genes (**Figure 5F**). By intersection genes obtained from three machine learning methods (**Figure 5G**), we identified five key genes: DUOX1, CD36, AGTR1, FHL5, and SSR4.

**Figure 6:**
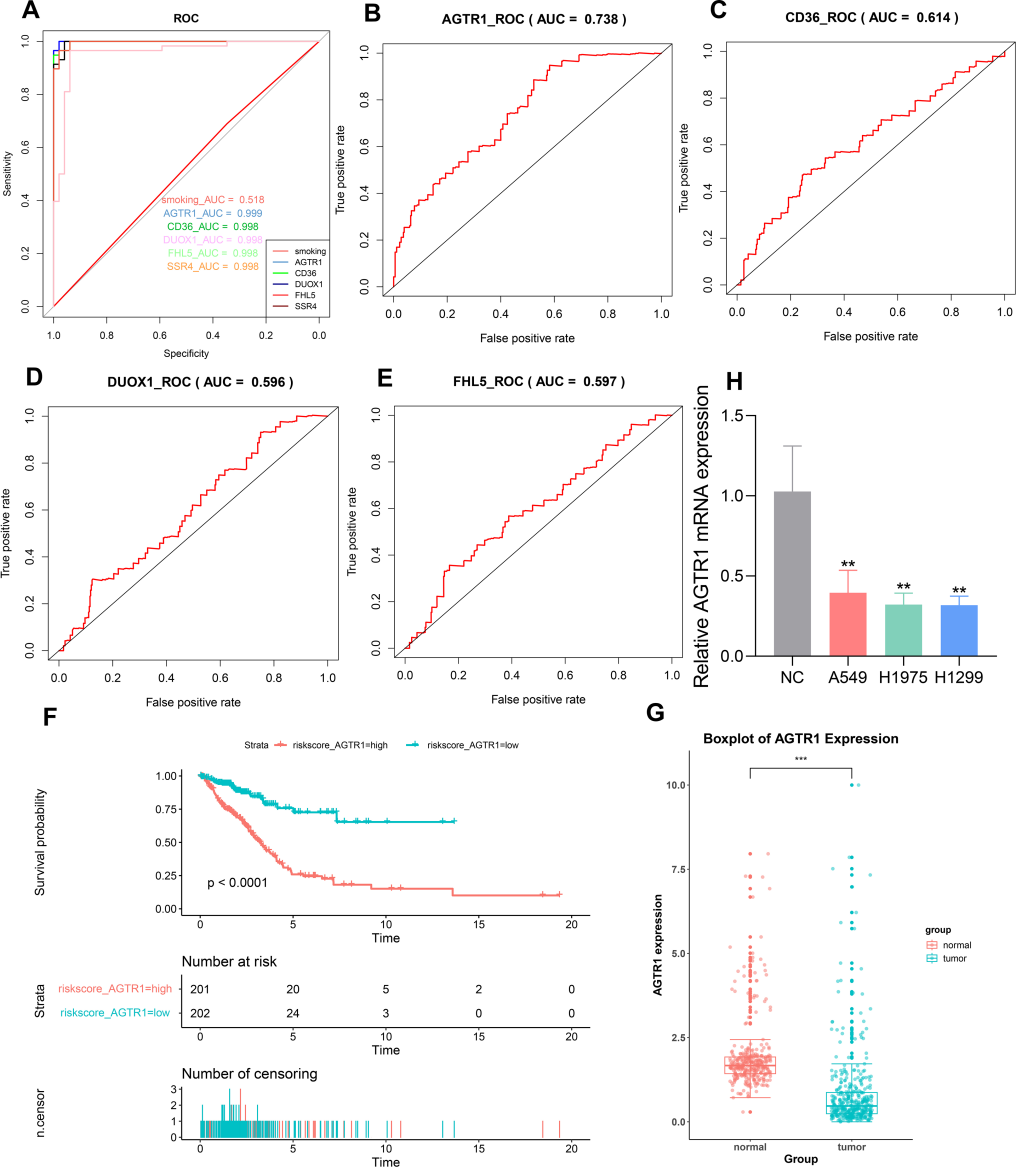
Screen the key genes with the highest correlation to LUAD through ROC curve, Cox regression analysis and box plot. ROC curve to validate the ability of predicting key genes **(A)**. Generated ROC curves using a risk scoring table for AGTR1, CD36, DUOX1, and FHL5 **(B–E)**. Survival curve for AGTR1 **(F)**. Box plot was used to describe the difference in expression levels of AGTR1 between LUAD samples and normal samples, *** p < 0.001 vs. normal group. **(G)**. The mRNA level of AGTR1 in NC group and LUAD cell lines (A549, H1975, H1299), ** p < 0.01 vs. NC group **(H)**.

To evaluate the reliability of the key genes identified by machine learning methods, we plotted ROC curves. The ROC curves of AGTR1, CD36, DUOX1, FHL5 and SSR4 yielded excellent AUCs of 0.999, 0.998, 0.998, 0.998, and 0.998, respectively (**Figure 6A**). When the AUC value under the ROC curve is closer to 1, it indicates a stronger correlation between the gene and LUAD. In addition, as a key clinical factor inducing LUAD, smoking only showed an AUC value of 0.518, which is much lower than the AUC values of the five key genes. By conducting Cox regression analysis based on clinical data obtained from TCGA database, key genes that exert a significant influence on the survival and prognosis of LUAD were verified. We obtained the risk score table through Cox univariate regression analysis on five key genes, in which the p value of SSR4 was found to be greater than 0.05, which was not statistically significant. Then we plotted the ROC curves related to survival for the other four key genes. The AUC values of AGTR1, CD36, DUOX1, and FHL5 were 0.738, 0.614,0.596, and 0.597, respectively (**Figures 6B–E**). On account of the AUC values, we have identified AGTR1 as the key gene for further analysis. Then, based on the risk score table, survival curve of AGTR1 was plotted. When the survival time was the same, the survival probability of the low-risk group was higher (**Figure 6F**). A box plot was plotted based on sample information acquired from TCGA to represent the differential expression of AGTR1 between normal and LUAD samples, showing that AGTR1 was significantly downregulated in the tumor group (**Figure 6G**). Subsequently, the qRT-PCR results showed that the expression of AGTR1 is significantly lower in the LUAD group (A549, H1299 and H1975) compared to the NC group (**Figure 6H**).

### 3.5 Infiltration of immune results

**Figure 7:**
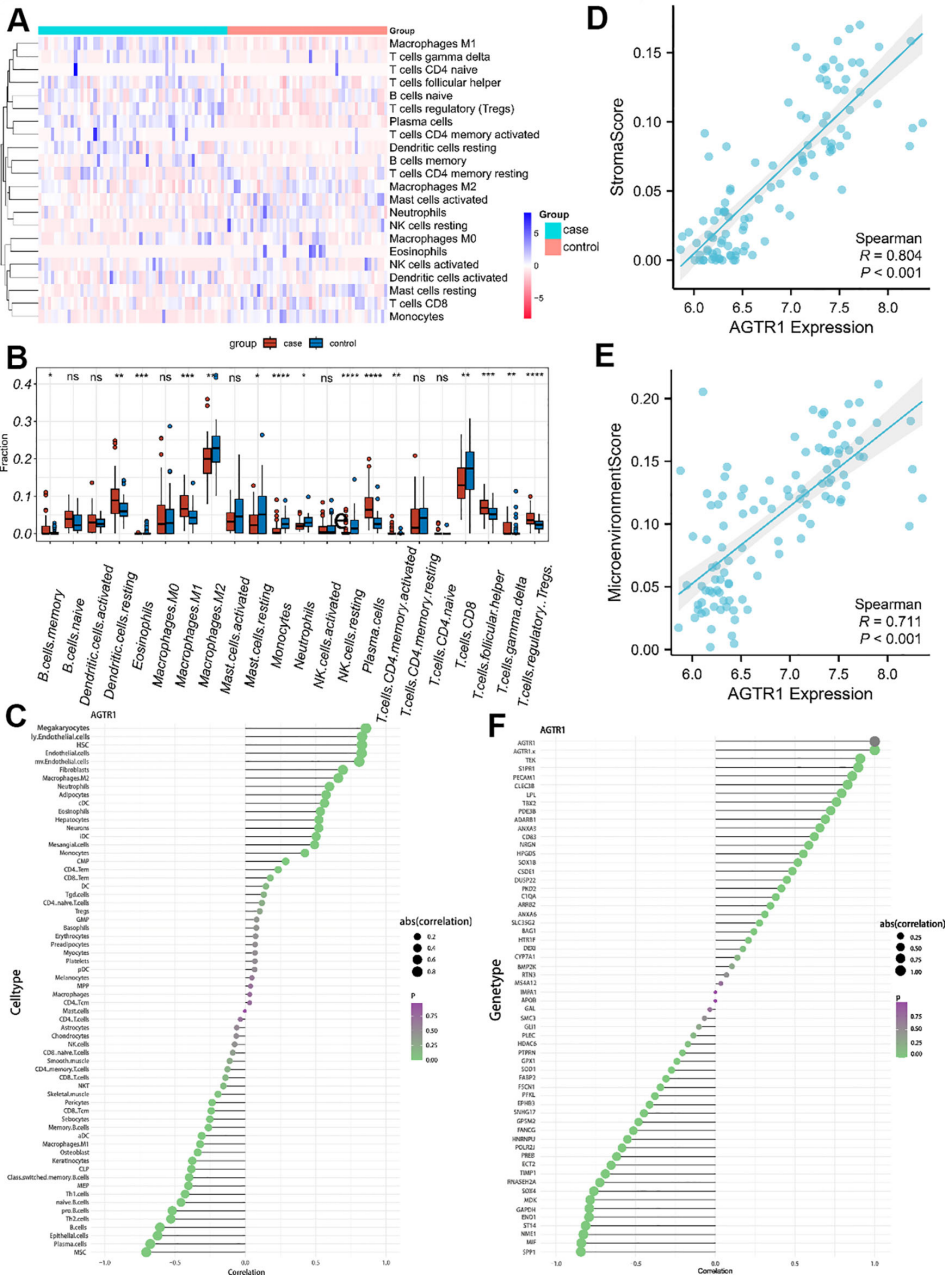
Infiltration of immune cells results. The differences in the abundance of immune cells in different groups **(A)**. Relative cellular fraction of 22 immune cell in LUAD and normal samples by Cibersort, * p < 0.05 vs. control group, ** p < 0.01 vs. control group, *** p < 0.001 vs. control group, **** p < 0.0001 vs. control group, nc is p > 0.05 vs. control group. **(B)**. The lollipop plots showed the association between AGTR1 and different immune cell **(C)**. Scatter plots are used to show the association of AGTR1 with immune stroma and microenvironment **(D, E)**. Among them, “Stromal score” refers to the abundance score of stromal cells in the tumor microenvironment **(D)**, the “Microenvironment score” refers to the abundance score of all types of cells in the tumor microenvironment, including immune cells, stromal cells, tumor cell and so on **(E)**. The lollipop plots showed the correlation of AGTR1 with immune chemokines and immune regulatory factors **(F)**.

CIBERSORT was utilized to describe immune infiltration trend of immune cells. To ulteriorly investigate the distinction in immune cell types between LUAD and normal samples, we estimated the abundance of immune cells based on the gene expression matrix obtained from GSE10072. Compared to normal samples, LUAD samples showed a higher abundance of memory B cells, resting dendritic cells, eosinophils, M1 macrophages, plasma cells, CD4 memory activated T cells, follicular helper T cells, gamma-delta T cells and regulatory T cells. In contrast, M2 macrophages, resting mast cells, monocytes, neutrophils, resting natural killer cells and CD8 T cells were under expressed in LUAD samples. Correlation heatmap (**Figure 7A**) and Boxplot (**Figure 7B**) were employed to show the differences in the abundance of immune cells from different groups. What’s more, we showed the relativity between AGTR1 and immune cells through lollipop plot (**Figure 7C**). Among them, megakaryocytes, M2 macrophages, and neutrophils showed positive correlation with the expression level of AGTR1, while plasma cells and B cells showed negative correlation with the expression level of AGTR1. Afterwards, the scatter plots showed a strong correlation of AGTR1 with immune stroma (R=0.804, P<0.001) (**Figure 7D**) and immune microenvironment (R=0.711, P<0.001) (**Figure 7E**). Immune chemokines and immune regulatory factors analysis showed that, the expression levels of S1PR1, PECAM1, and ADARB1 were positively associated with AGTR1, while the expression levels of SPP1, ENO1, and ECT2 were negatively related to AGTR1 (**Figure 7F**). Immune checkpoint analysis showed that the expression levels of STXBP6, PECAM1, and ADARB1 were positively associated with AGTR1, while the expression levels of SPP1, ENO1, and ECT2 were negatively related to AGTR1 (**Supplementary Figure S1**).

### 3.6 Construction of a ceRNA network

**Figure 8:**
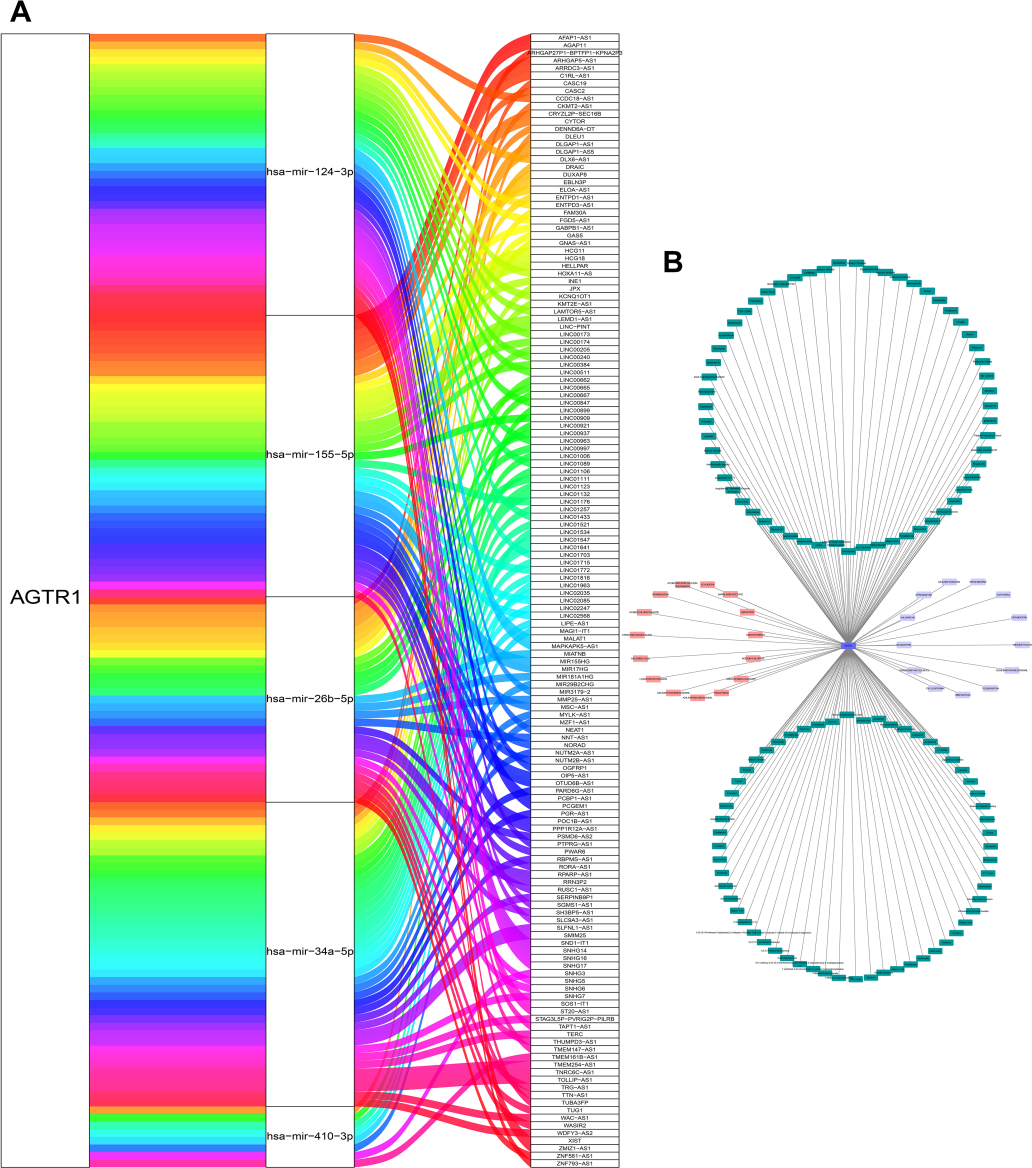
Construction of CeRNA network and acquisition of potential drugs. The ceRNA network constructed by AGTR1 and its related miRNA and lncRNA **(A)**. The drug-gene network of AGTR1. Drugs marked in pink obtained from DGIdb database, drugs marked in green obtained from CTD database, and the drugs marked in purple were overlapping targeted drugs in two databases **(B)**.

We built a ceRNA regulatory network to explore the regulatory mechanisms of AGTR1. The miRTarBase database was used to predict miRNAs related to AGTR1 and finally acquired 6 miRNAs (hsa-miR-34a-5p, hsa-miR-155-5p, hsa-miR-410-3p, hsa-miR-155-3p, hsa-miR-26b-5p, hsa-miR-124-3p). Afterwards, based on the miRNAs obtained above, relevant lncRNAs were identified using the miRNet database. The lncRNAs could be found for 5 miRNAs (hsa-miR-34a-5p, hsa-miR-155-5p, hsa-miR-410-3p, hsa-miR-26b-5p, hsa-miR-124-3p), and 149 pairs of miRNA-lncRNAs were obtained at last (**Figure 8A**).

### 3.7 Potential drugs of key gene

To seek potential drugs for treating LUAD, we searched the DGIdb and CTD databases for potential drugs targeting AGTR1.15 and 118 drugs targeting AGTR1 were discovered in these databases (**Figure 8B**), respectively. There are a total of 13 overlapping targeted drugs included Cyclosporine, Candesartan, Benazepril, Valsartan, Eprosartan, Dexamethasone, Nitrendipine, Captopril, Perindopril, Indomethacin, Olmesartan Medoxomil, Telmisartan and Irbesartan in two databases.

## 4 Discussion

Lung cancer is one of the types of cancer with the highest mortality rate, with the LUAD subtype accounting for about 50% of all lung cancer deaths (17). Although there has been some progress in LUAD treatment for the past few years, the general survival rate of LUAD patients is still significantly bleak. Therefore, it remains crucial to delve into therapeutic targets aimed at enhancing the survival rate of LUAD patients. Furthermore, studying the patterns of cell invasion associated with LUAD immunity can help us understand how LUAD affects the immune system. In this study, we constructed co-expression gene modules and used machine learning methods to screen the key genes associated with LUAD. The reliability of key genes was assessed through functional enrichment, ROC analysis, and COX regression analysis. QRT-PCR further revealed differences in the expression of key genes in LUAD and normal cells. In addition, immune infiltration analysis was utilized to understand the interaction between LUAD and the immune response, as well as the relationship between key genes associated with LUAD and immune response. A CeRNA network was created to explore the regulatory mechanisms of key genes and we further sought the potential drug targets.

With the development of high-throughput sequencing technology, many bioinformatics algorithms and public databases provide analytical tools and scientific basis for exploring cancer treatment and finding potential therapeutic targets (15). Through the analysis of gene expression data from LUAD group and normal group based on GSE10072, we found 631 DEGs, including 206 upregulated and 425 downregulated genes. Furthermore, 9 hub modules highly associated with LUAD were obtained by WGCNA. We further obtained 5 key genes (DUOX1, CD36, AGTR1, FHL5, and SSR4) by applying RF, Lasso and SVM-RFE based on the intersection genes from DEGs and hub module genes. Among the above five key genes, DUOX1 (log2FC=-2.1765235), CD36 (log2FC=-2.4169837), AGTR1 (log2FC=-1.6991027) and FHL5 (log2FC=-1.1938571) were significantly downregulated gene (log2FC<-1), while SSR4 (log2FC=1.0115043) was significantly upregulated gene (log2FC>1), which indicated a potential correlation of these 5 key genes with LUAD. Subsequently, the five key genes were validated to exist in salmon module (cor=0.97, p<1e-200), which was the module with the highest correlation with LUAD among the 9 modules obtained from WGCNA. The Module Memberships of five key genes in salmon module were greater than 0.7, among them AGTR1, CD36, FHL5 and DUOX1 had Module Membership greater than 0.8, which demonstrated the crucial role of the five key genes in salmon module. AGTR1, CD36, FHL5 and DUOX1 had gene significance greater than 0.8 in lung tumor, while SSR4 had gene significance greater than 0.7, which revealed that these key genes were strongly associated with LUAD.

Then, further validation was conducted on the 5 key genes. The ROC results revealed that the AUC values of the five key genes are all greater than 0.9, which indicated a strong correlation between key genes and LUAD. We also compared the effects of smoking and 5 key genes on LUAD using ROC analysis, and the results revealed a higher relationship between the 5 key genes and LUAD. Subsequently, we performed Cox univariate regression analysis on five key genes and their risk scores were calculated, which suggests that DUOX1, CD36, AGTR1, FHL5 are risk factors for survival and prognosis. Next, their ROC curves were plotted. Among them, AGTR1 had the largest AUC value of 0.738 among the four key genes, and the AUC values of the other three genes were all less than 0.7, which reveals that AGTR1 is a key feature of survival and prognosis in LUAD patients. Further, according to the risk score, the survival curve of AGTR1 indicated that the high-risk group exhibited notably greater mortality compared to the low-risk group. In addition, AGTR1 was also significantly down-regulated in LUAD samples obtained from TCGA. The qRT-PCR results showed that AGTR1 was downregulated in A549, H1975, and H1299 cell lines. A549, H1975, and H1299 cell lines were derived from tumor tissues of LUAD patients with different degrees of metastasis and genders, which indicated that AGTR1 is downregulated in LUAD cells with different genetic backgrounds. So AGTR1 was identified as a key gene for further analysis in this study.

Subsequently, enrichment analysis showed that the intersection genes of DEGs and hub module genes were mainly related to response to hormone, circulatory system process, vasculature development, pathways in cancer, Relaxin signaling pathway, GPCR ligand binding, signaling by GPCR, metabolism of lipids, innate immune system and so on. Cancer cells are “transformed cells” with a series of genetic and epigenetic mutations that enable them to self-renew, proliferate, lose control of apoptosis, migrate, invade, and ultimately form tumors (18). Abnormal activation of pathways in cancer can lead to abnormal proliferation and dysregulation of apoptosis control in LUAD cells (19). In this process, abnormal lipid metabolism may provide the necessary energy and metabolic substrates for the growth, invasion, and metastasis of LUAD cells. Recent studies have found that accumulation of iron-dependent lipids through peroxidation can cause regulated cell death which is associated with the occurrence of LUAD (20). During the process of proliferation and migration of tumor cells, oxygen and nutrients are also necessary substances for the development of LUAD, which rely on vasculature development and circulatory system process. They can not only provide sufficient oxygen and nutrients for LUAD cells from surrounding tissues, but also provide migration channels for tumor migration (21). During the occurrence of LUAD, cancer stem cells (CSCs) can effectively invade blood vessel lumen and survive in circulation. CSCs migrate to other sites through the bloodstream, and then de-differentiate into CSCs with tumorigenic potential (22). Hormone response also plays a crucial part in LUAD cell proliferation and invasion. Researches has shown that estrogen can upregulate osteopontin (OPN) expression and promote lung cancer cell migration and growth by activating the MEK/ERK signaling pathway through Estrogen Receptor Beta (ERβ) (23, 24). Moreover, INSL4, a member of the relaxin family, promotes the proliferation and aggressiveness of LUAD cells by upregulating the MAPK and AKT signaling pathways (25). In addition, during the proliferation and invasion of tumors, the immune system plays a crucial defensive role by detecting and eliminating abnormal cells, including LUAD cells, while abnormal immune recognition promotes tumor development. GPCR ligand binding and signaling by GPCR are currently a hot topic in cancer immunology research, and some members of GPCR have been found to act as prognostic factors in diversified cancers. By activating downstream genes in GPCR ligand binding and signaling by GPCR, immune infiltration and anti-tumor effects of immune cells can be promoted in LUAD tissues (26). In addition, GPCRs have several key functions including regulation of cell motility, growth, and differentiation, which also have an impact on LUAD development (27). In summary, the intersection genes take part in the proliferation and migration of LUAD cells, and also participate in immune processes, which showed a high correlation with LUAD.

Further enrichment analysis reveals that AGTR1 has a strong correlation with LUAD. AGTR1 is a member of the seven-transmembrane-spanning G-protein coupled receptor superfamily. Multiple studies showed that the renin-angiotensin system (RAS) plays an important role in lung cancer (28). AGTR1 gene is an important component of the RAS system, which can encode the angiotensin II receptor type 1 (AT1R). High expression of AGTR1 has been found to be linked with less lymph node metastasis and mesenchymal-epithelial transition factor (MET) mutations. It is also related to the anti-tumor immune microenvironment characterized by immune cell infiltration (28). In addition, AGTR1 participates in pathways of GPCR ligand binding and signaling by GPCR to mediate cell growth and proliferation (27). These results suggest that AGTR1 may impact on the occurrence and development of LUAD through multiple pathways.

The relationship between immune cells and LUAD was described using immune infiltration analysis. The results of this study showed that compared to normal samples, LUAD samples exhibited higher abundance of memory B cells, plasma cells and resting dendritic cells. In contrast, M2 macrophages, neutrophils and resting natural killer cells were under expressed in LUAD samples. The involvement of immune dysfunction in the pathogenesis of lung cancer has been extensively studied (29). Memory B cells and plasma cells both originate from activated B cells and play a significant role in immune defense in the body. Memory B cells are key reservoirs for plasma cell generation (30). Memory B cells store the information of antibodies produced after the first contact with antigens. Once they encounter the same pathogen again, memory B cells can rapidly differentiate into plasma cells and produce a large number of antibodies to cope with pathogen invasion, thus achieving the effect of immune protection (31). This mechanism plays an important role in LUAD. Dendritic cells (DC cells) recognize pathogens in the innate immune system and activate immune cells in the adaptive immune system (32, 33). DC cells can detect homeostatic imbalances, and process antigens to present them to T cells for activating T cell responses (2, 29). Macrophages are a major type of immune cell that is divided into M1 and M2 types. Among them, M2 macrophages are often involved in the repair and recovery of wound or inflammatory sites (34). M2 macrophages can participate in anti-inflammatory responses and repair damaged tissues. They can be activated by immune complexes, TLR ligands, glucocorticoids, or IL-10, to exert their anti-inflammatory functions and alleviate tissue damage (35). Neutrophils, a form of white blood cell, are the body’s initial defense against infections. They can quickly reach the site of infection or inflammation and kill pathogens through immune stress such as phagocytosis and digestion (36). The function of neutrophils is relatively complex. These cells can not only promote lung cancer carcinogenesis through angiogenesis and metastasis, but also limit tumor growth by producing anti-tumor and cytotoxic mediators (29). Natural killer cells (NK cells), a type of lymphocytes, have a crucial role in defending the body against viral infections and tumor cells (37). When NK cells come into contact with target cells, they send toxin vesicles into the target cells through cell spikes, which causes the target cells to dissolve. In addition, NK cells also secrete IFN-γ, IL-2, and other multiple cytokines, which regulate and promote the activities of other immune cells, thereby improving immune response and inhibiting tumor development (38). In summary, our research results demonstrate that memory B cells, plasma cells and resting dendritic cells play a normal defensive role in LUAD, and the immune response of M2 macrophages, neutrophils and NK cells to the tumor is suppressed, which promoted the progression of LUAD.

Further correlation analysis revealed that AGTR1 exhibits a strong correlation with the immune stroma, immune microenvironment, and immune cells. In immune cells, AGTR1 exhibited a positive correlation with M2 macrophages and neutrophils, while displaying a negative correlation with memory B cells and plasma cells. These immune cells that are correlated with AGTR1 are consistent with those that exhibit abundance differences between the case and control groups. As a receptor for angiotensin II (Ang II), which is a biologically active substance produced by a part of RAS, AGTR1 can activate macrophages by binding with Ang II, resulting in the production of inflammatory mediators such as cytokines and chemical mediators. This leads to exacerbated inflammatory response and triggers immune reactions, ultimately inhibiting tumor formation (39). Ang II is also present in neutrophils, and when it binds with AGTR1, it can enhance the activity of neutrophils, thus eliminating tumor cells (40). In conclusion, AGTR1 can regulate the macrophages and neutrophils through its ligand Ang II, thereby influencing the occurrence and development of LUAD. Our research indicates that downregulation of AGTR1 leads to a decrease in M2 macrophages and neutrophils, suppressing their immune functions and facilitating the progression of LUAD. However, memory B cells and plasma cells still exhibit high expression in LUAD, which suggesting that AGTR1 does not affect the activation of memory B cells and plasma cells, and their role in tumor immunity. Further validation through immune chemokine, immune checkpoint and immune regulatory factors analysis revealed that AGTR1 shows a positive correlation with PECAM1 and ADARB1, and a negative correlation with SPP1 and ENO1. Platelet endothelial cell adhesion molecule 1 (PECAM1), also named CD31, is a differentiation antigen expressed on the surface of granulocytes, monocytes, and platelets (41). Research has shown that PECAM1 expression is positively correlated with neutrophils and macrophages. PECAM1 can also regulate the expression of vascular endothelial growth factor (VEGF), which plays a crucial part in the development of LUAD (42). Adenosine deaminase RNA-specific B1 (ADARB1), also named ADAR2, is an adenosine-to-inosine (A-to-I) RNA editing enzyme that has been found to play a crucial role in the development of cancer. Research suggests that according to research, there is a positive correlation between ADARB1 and NK cell expression in cancer, which plays a part in inhibiting the development and metastasis of LUAD (43, 44). Secreted phosphoprotein-1 (SPP1), also known as osteopontin. It is expressed in macrophages and enhances the migratory and invasive capabilities of LUAD cells by upregulating the expression of COL11A1. SPP1 is also enriched in cell adhesion, PI3K-Akt signaling pathway, and ECM-receptor interaction in the study of LUAD, which reveals that SPP1 plays an important role in LUAD (42, 45). Enolase 1 (ENO1), also known as alpha-enolase, is a multifunctional cancer protein which is widely expressed in multiple cell types. The glycolytic function of ENO1 is involved in relieving cell energy regulation, maintaining tumor proliferation, and inhibiting apoptosis of cancer cells. ENO1 can also induce regulatory T cells (Tregs) to promote cancer development by suppressing anti-tumor immune responses. Additionally, ENO1 may mediate the PI3K/AKT pathway and its downstream signaling pathways to affect tumor cell activity (46). Therefore, our study showed that downregulation of AGTR1 could downregulate the expression of PECAM1 and ADARB1, which suppresses the immune functions of neutrophils, macrophages, and NK cells, while upregulating the expression of SPP1 and ENO1, which promotes the immunosuppressive function of Tregs cells. Furthermore, the dysregulation of PECAM1 and SPP1 affects cell migration, which promoted the development of LUAD. Nevertheless, the specific regulatory mechanisms of AGTR1 on PECAM1, ADARB1, SPP1, and ENO1 have not been discovered yet and require further investigation.

In summary, in our study, based on WGCNA, machine learning methods, ROC and COX analysis, the key gene related to LUAD, AGTR1, was identified. Further functional enrichment and immune analysis revealed that AGTR1, as a receptor of Ang II, inhibits the growth and proliferation of LUAD cells through GPCR ligand binding and signaling by GPCR. Additionally, AGTR1 also activates M2 macrophages and neutrophils by binding with Ang II, which is crucial for tumor immune response. Further analysis reveals a strong correlation between AGTR1 and immune checkpoints, as well as immune chemokines. Hence, downregulation of AGTR1 in LUAD leads to immune suppression and it also promotes the growth and proliferation of LUAD cells, which facilitating the development of LUAD. Based on these findings, AGTR1 is considered to be an important biomarker in LUAD and a potentially valuable therapeutic target for further research.

Next, to gain deeper insights into the regulatory mechanism of AGTR1 on LUAD, we constructed a CeRNA regulatory network. In this study, we identified six miRNAs associated with AGTR1. Current studies have shown that as a tumor suppressor, has-miR-124-3p may inhibit the progression of a variety of tumors, including NSCLC (47, 48). Has-miR-155-5p is up-regulated in LUAD tissues, which may affect the occurrence and prognosis of LUAD (49). In addition, hsa-miR-34a-5p can induce cell cycle arrest and apoptosis by regulation of p53, and can participate in the tumorigenesis of prostate cancer cells through the SIRT1/TP53 axis (50). Has-miR-410-3p has anti head and neck squamous cell carcinoma effects (51). But the role of these miRNAs on LUAD is unclear. Moreover, we also reversely predicted lncRNA associated with microRNA. LncRNA HCG18 has been reported to act as an oncogene in LUAD and enhance LUAD development by targeting the miR-34a-5p/HMMR axis (52). LncRNA TTN-AS1 can facilitate the malignant progression of LUAD by regulating miR-142-5p/cyclin-dependent kinase 5 signaling pathway (2, 13). Some studies indicate that targeting PCBP1-AS1 enhances the therapeutic responsiveness of enzalutamide resistant cancers and reduces metastasis in LUAD (13, 53). In conclusion, the ceRNA network further confirmed the influence of AGTR1 on the occurrence and prognosis of LUAD.

In addition, we also predicted potential effective therapeutic drugs for LUAD. We obtained 13 overlapping potential agents targeting AGTR1 from the DGIdb and CTD databases, all of which have been used in the clinic. Cyclosporin can reduce secondary brain injury by inhibiting mitochondrial permeability transition (54). Candesartan, Benazepril, Valsartan, Eprosartan, Olmesartan Medoxomil, Telmisartan, Irbesartan, Captopril and Perindopril are commonly used angiotensin-converting enzyme inhibitor (ACEI) or angiotensin receptor antagonist (ARB), which can be used to treat cardiovascular diseases such as hypertension and heart failure (55–63). What’s more, Nirendipine can be combined with ACE to treat diseases such as hypertension (64). Indomethacin can be used to improve recovery in patients with traumatic brain injury (65). Among them, Candesartan, Benazepril, Valsartan, Epsartan, Olmesartan Medoxomil, Telmisartan, and Irbesartan have relatively minor side effects, including headache, dizziness, and hyperkalemia. They usually do not cause dry cough and are more tolerable for chemotherapy patients. Therefore, they may become a promising potential chemotherapy drug for treating LUAD by targeting AGTR1. However, no relevant studies on LUAD have been found for these drugs. Our findings may provide a basis for exploring effective drugs to treat LUAD.

## 5 Conclusions

In this study, five key genes, AGTR1, CD36, DUOX1, FHL5 and SSR4, associated with LUAD were identified through WGCNA and three machine learning methods. Furthermore, AGTR1 is considered as a key gene through ROC, COX and qRT-PCR analysis. Functional and immune analysis revealed that AGTR1 is strongly associated with hormone response and circulatory system, and can inhibit the development of LUAD by regulating cell growth and proliferation. In addition, AGTR1 may well exert inhibitory effects on LUAD by regulating immune cells, immune chemokines, immune checkpoints, and immune regulatory factors. Therefore, AGTR1 can be a novel potential biomarker for diagnosis and treatment of LUAD. However, the pathogenic molecular mechanism of AGTR1 in LUAD needs to be further explored through cell, animal, and clinical experiments, which helps us further clear out the role of AGTR1 in LUAD and investigate the pathogenesis of LUAD.

## Data Availability

Publicly available datasets were analyzed in this study. This data can be found here: GSE10072 from GEO (https://www.ncbi.nlm.nih.gov/geo/query/acc.cgi?acc=GSE10072) and TCGA (https://portal.gdc.cancer.gov/) databases.
